# Diagnostic Challenges in Creutzfeldt-Jakob Disease: A Case Report of an Atypical Presentation

**DOI:** 10.7759/cureus.84233

**Published:** 2025-05-16

**Authors:** Shafaq Ismail, Mohamed Shafei, Jahanzeb Rehan

**Affiliations:** 1 Acute Medicine, Sherwood Forest Hospitals NHS Foundation Trust, King's Mill Hospital, Mansfield, GBR; 2 Respiratory Medicine, Sherwood Forest Hospitals NHS Foundation Trust, King's Mill Hospital, Mansfield, GBR; 3 Stroke Medicine, Sherwood Forest Hospitals NHS Foundation Trust, King's Mill Hospital, Mansfield, GBR

**Keywords:** creutzfeldt-jakob disease, diagnostics, neurodegenerative disorder, prion disorder, rt-quic

## Abstract

Creutzfeldt-Jakob disease (CJD) is a rare, fatal neurodegenerative prion disorder that often mimics other neurological conditions, including stroke. This case report highlights the diagnostic challenges in a patient with CJD initially presenting with nonspecific neurological symptoms.

A 70-year-old male with a history of bladder cancer, hypertension, osteoarthritis, and migraines presented with acute confusion, fever, and nonspecific symptoms. Initial evaluation revealed altered mental status, elevated inflammatory markers, and chest consolidation on computed tomography (CT) imaging. Despite negative blood cultures and cerebrospinal fluid (CSF) analysis, the patient's condition deteriorated rapidly. The patient exhibited acute confusion worsening over two days, bilious vomiting, fever (39.1°C), generalized abdominal pain, persistent nausea, and decreased eating, drinking, and mobility. Initial differentials included posterior-circulation stroke, hospital-acquired pneumonia, and urinary tract infection.

Blood and urine cultures, CT of the head, and CT pulmonary angiography were inconclusive. Magnetic resonance imaging (MRI) of the head revealed bilateral temporo-parietal cortical restricted diffusion, prompting neurological evaluation. The final diagnosis was confirmed by a positive real-time quaking-induced conversion (RT-QuIC) polymerase chain reaction (PCR) test for CJD. Key diagnostic findings included clear colorless CSF with positive RT-QuIC PCR, elevated C-reactive protein (281 mg/L) and white cell count (15.2 x 10ˆ9/L) with neutrophilia (14.1 x10ˆ9/L), and MRI showing bilateral temporo-parietal cortical restricted diffusion.

This case underscores the importance of considering CJD in patients presenting with atypical neurological symptoms, even when initial presentations suggest more common conditions like stroke. Early recognition, appropriate neuroimaging, and specialized tests like RT-QuIC are crucial for the timely diagnosis and management of this rare but devastating illness.

## Introduction

Creutzfeldt-Jakob disease (CJD) is a rare disease, with one case in a million people per year [1]. CJD is a part of a group of diseases called prion diseases, wherein there is misfolding of proteins in the central nervous system. It is known to be uniformly fatal. It manifests with neurodegeneration, presenting with rapidly progressive dementia and motor dysfunction. Motor dysfunction can range from involuntary myoclonus to signs of cerebellar involvement, including lack of coordination and problems with walking and balance, as well as visual disturbances involving blindness in later stages. Signs include both pyramidal and extra-pyramidal presentations, including spasticity, rigidity, dyskinesia, and frontal release [2,3].

CJD includes four main subtypes: sporadic, familial, variant, and iatrogenic. About 85% of presenting cases are sporadic. This generally occurs in cases of already existing prion proteins misfolded for unknown reasons. This results in interference with the brain's functions. Sporadic CJD can be further divided into definite, probable, and possible CJD. It is thought to be definite when it is diagnosed through standard neuropathological techniques, such as immunocytochemistry, Western blot confirmed protease-resistant PrP, and/or presence of scrapie-associated fibrils. CJD is probable if there is evidence of neuropsychiatric disorder plus positive real-time quaking-induced conversion (RT-QuIC) test (both present in our patient) or there is rapidly progressive dementia and at least two out of the four clinical features, including myoclonus, visual or cerebellar signs, pyramidal/extrapyramidal signs, and akinetic mutism, as well as a positive result on at least one of the laboratory tests: a typical EEG (periodic sharp wave complexes) during an illness of any duration, a positive 14-3-3 protein cerebrospinal fluid (CSF) assay in patients with a disease duration of less than two years, high signal in caudate/putamen on magnetic resonance imaging (MRI) brain scan or at least two cortical regions (temporal, parietal, occipital) either on diffusion-weighted imaging (DWI) or fluid attenuated inversion recovery (FLAIR), and without routine investigations indicating an alternative diagnosis.

Possibility of CJD arises when there is progressive dementia and at least two out of these four clinical features of myoclonus, visual or cerebellar signs, pyramidal/extrapyramidal signs, or akinetic mutism, along with absence of a positive result for any of the four tests above that would classify a case as "probable", with duration of illness less than two years and without routine investigations indicating an alternative diagnosis [4].

Of CJD cases, 5-15% occur as a result of inheritance of a mutated (change of DNA sequence in a gene) prion protein gene, namely, PRNP (prion protein gene). This is known as familial CJD and occurs when a person with CJD also has an affected first-degree relative. Variant subtypes pertain to those individuals who contract the disease from eating contaminated beef. It is linked to eating beef from cattle infected with mad cow disease (also called bovine spongiform encephalopathy) [5]. Cannibalism is also a risk factor. Lastly, iatrogenic cases occur in case a person comes into contact with prions in a healthcare setting or due to biological products. This can include people receiving a transplant of biologic products. Examples include corneal or dura mater grafts. In case the donor suffered from CJD, but was not discovered prior to donation, recipients can contract CJD [6].

This study aimed to present a 70-year-old man who initially presented with symptoms of likely an infection of uncertain origin but was ultimately diagnosed as a case of CJD. Through this study, we have brought to light the importance of having a high index of suspicion for CJD. While it may be rare, its incidence is still on an upward trend, and it is worthwhile to have it among the list of differentials for early detection.

## Case presentation

Here, we present a case of a 70-year-old Caucasian male patient who presented with a combination of symptoms and was a diagnostic conundrum for clinicians. Initially, his symptoms were vague and pointed toward a likely infection; however, ultimately, he was diagnosed as a case of CJD. Due to indistinct and overlapping symptoms, several investigations were done, and a multidisciplinary approach was taken. He presented with a two-day history of acute confusional state that was worsening, along with one episode of bilious vomiting and high-grade fever (39.1°C). On collateral history, his family reported occasional cough, generalized abdominal pain, persistent nausea, and decreased eating, drinking, and mobility.

He had been recently discharged from the hospital after being treated for urosepsis. He had a background of transurethral resection of bladder tumor (TURBT) secondary to urinary bladder cancer (two years ago), hypertension, osteoarthritis, and migraines. He was an ex-smoker who quit six months ago. Regular medications included anti-hypertensives and migraine prophylaxis. Before this acute onset of confusion, he was known to be independent with his activities of daily living.

On presentation to the hospital by ambulance, his observations showed a high temperature, along with mildly elevated blood pressure (173/98), tachypnoea (24/minute), and low oxygen saturation (86%), for which he was given oxygen (2.5 L). Examination revealed a Glasgow Coma Scale (GCS) score of 14/15 (E4, V4, M6), left-sided crackles on chest auscultation, and suprapubic tenderness. Posterior-circulation stroke was considered owing to acute altered mental status; however, due to recent hospital admission, hospital-acquired pneumonia (HAP) was also a possibility. Nonetheless, due to the recent diagnosis of urosepsis, urinary tract infection (UTI) was also among the differentials.

Blood tests revealed markedly raised inflammatory markers, including C-reactive protein (CRP) at 281 mg/L (normal: <5), white cell count (WCC) of 15.2 x 10^9/L (normal: 4-10), and D-dimer at 19080 ng/ml (normal: <500), along with stage 1 acute kidney injury (AKI) and low magnesium (0.54 mmol/L) and folate (3.4 microgram/L) levels. While these low levels of folate and magnesium and underlying infection were thought to be a contributing factor for confusion, he was treated with intravenous (IV) antibiotics, and IV fluids and electrolyte replacement were given. Blood and urine cultures were negative. Computed tomography (CT) of the head did not show stroke, and CT pulmonary angiography (CTPA) was done to rule out pulmonary embolism (PE). However, it was inconclusive for PE but showed left lower zone consolidation.

Collateral history from the family was taken, and they reported no recent falls; however, he had been quite forgetful in the last six months. He was unable to tell time or write properly. He was struggling to get his words out and could not hold a conversation. They were unable to make out what he was saying. They also informed that his recent scans showed new bladder lesions.

This cognitive decline and speech disorder raised the possibility of central nervous system (CNS) pathology, including dementia, cerebrovascular disease, CNS infection, or metastatic brain disease.

The psychiatry team's input was requested. They examined the patient and assessed that he had been feeling suicidal over the past few months with a low mood. He had not shared his feelings with his family, and generally felt agitated sometimes. He had occasionally been struggling to sleep. This change in behavior raised concerns regarding cognitive decline.

**Figure 1:**
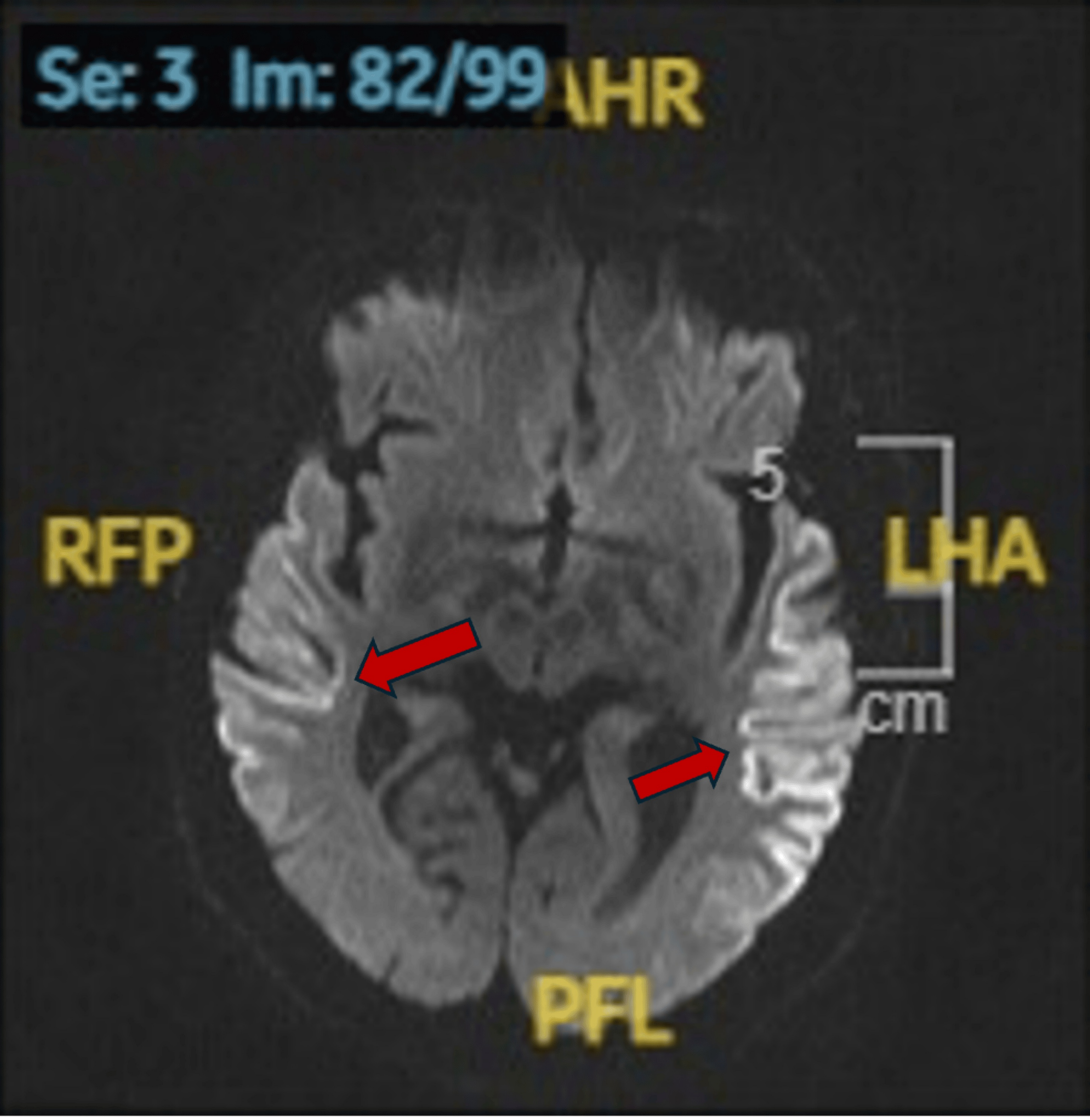
Axial section of diffusion-weighted images showing bilateral cortical ribboning pattern.

**Figure 2:**
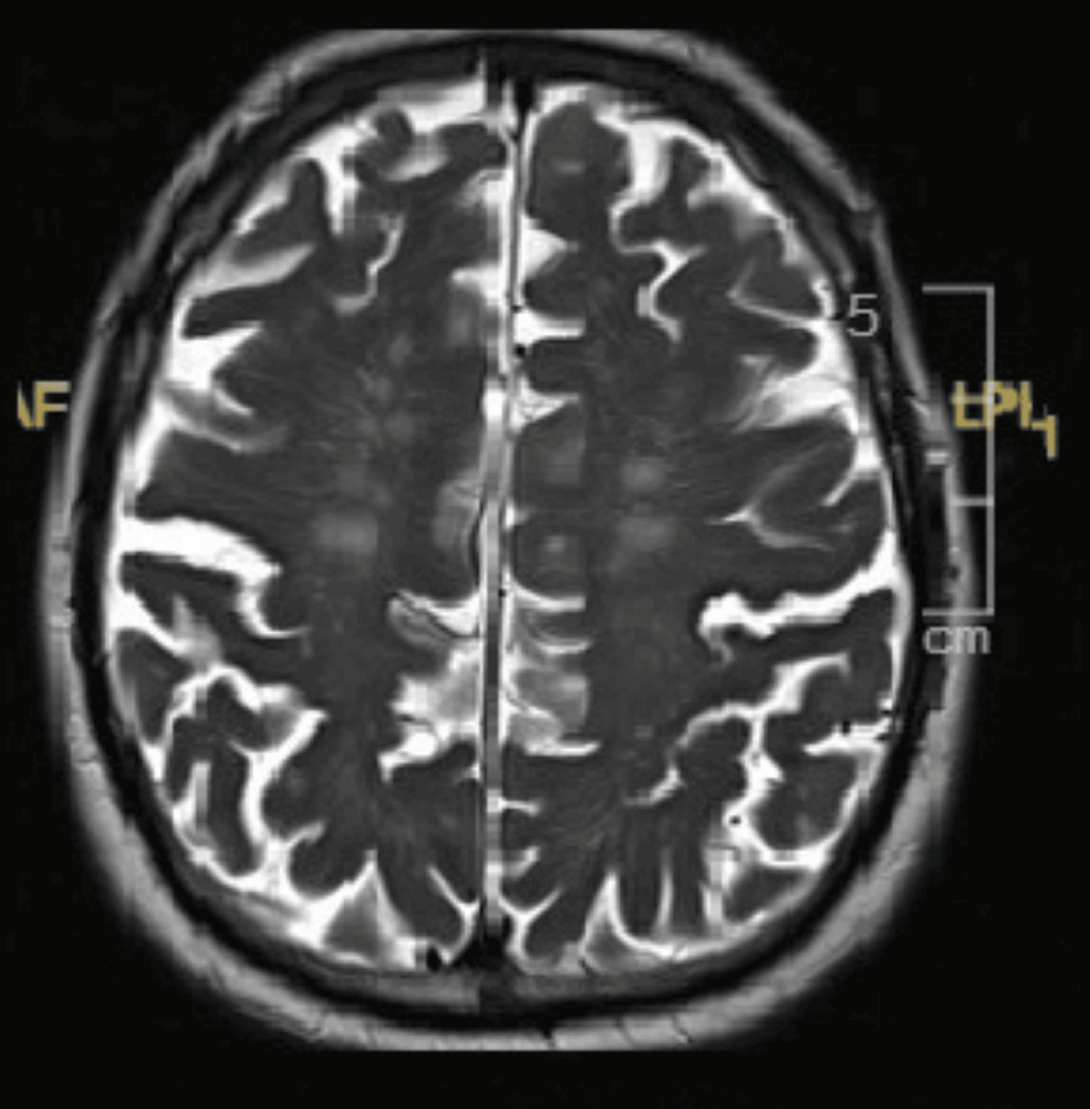
T2-weighted images showed mild diffuse cortical atrophy and small blood vessel ischemic changes.

Since confusion was ongoing, metastatic brain disease and acute ischemic stroke needed to be excluded; hence, magnetic resonance imaging (MRI) of the head was requested. It showed bilateral restricted diffusion in the temporo-parietal cortex with bilateral posterior cortical ribbon sign (Figures 1, 2). Keeping these findings in view, infarction, hypoxic ischemic encephalopathy (HIE), and CNS infection were the considerations. The stroke team was taken on board, and after reviewing, they ruled out stroke. Further, to rule out a CNS infection, lumbar puncture (LP) was done. The results were inconclusive initially (stated in Tables 1, 2).

**Table 1:** Investigations and findings (laboratory) RT-QuIC PCR: real-time quaking-induced conversion polymerase chain reaction.

Cerebrospinal fluid (CSF) tests	Findings (initial)	Findings (later)	Reference values
Cell count	Clear colorless fluid		
Red blood cells	3		Up to 5
Glucose	4		2.8-4.4 mmol/L
Culture	RT-QuIC PCR +		
Blood tests			
C-reactive protein (CRP)	281	4	<5 mg/L
White cell count (WCC)	15.2	7.3	4.0-11.0 x 10^9^/L
Neutrophils	14.1	6.6	1.5-8.0 x 10^9^/L
HIV and syphilis serology/paraneoplastic and encephalopathy antibodies	Negative		

**Table 2:** Investigations and findings (radiological).

CT head	There are areas of ill-defined hypoattenuation involving predominantly the white-matter in both occipital lobes, mainly the posterior periventricular regions. Conclusion: This finding could represent posterior reversible encephalopathy syndrome (PRES).
MRI head	Bilateral temporo-parietal cortical restricted diffusion. The differential diagnosis includes infarction, hypoxic-ischemic encephalopathy, infection, and metabolic.

The neurology team reviewed and asked for a few more tests, as paraneoplastic encephalitis, autoimmune encephalitis, and viral encephalitis were the only likely possibilities that were being contemplated. Blood samples that were sent for HIV and syphilis profile came back negative. Encephalopathy panel, including anti-leucine-rich glioma-inactivated 1 antibody (anti-LGI-1), contactin-associated protein-like 2 (CASPR2), N-methyl-D-aspartate receptor (NMDAR), gamma-aminobutyric acid receptors A/B (GABA-A/B), and α-amino-3-hydroxy-5-methyl-4-isoxazolepropionic acid 1/2 receptors (AMPA-1/2), and para-neoplastic antibodies (anti-Hu, Yo, Ri, Ma) were sent as well. When all these tests were inconclusive, one CSF sample was sent to the National CJD Research & Surveillance Unit (Edinburgh) to be tested for real-time quaking-induced conversion polymerase chain reaction (RT-QuIC PCR). This came back positive a few days later (Table 1). The patient was then declared positive for CJD. He was reviewed by the palliative team, who advised prescribing anticipatory medications for symptom control, as well as risperidone, as suggested by the psychiatry team. He was commenced on a fast-track pathway to discharge to a care home, where he passed away four months later.

## Discussion

CJD is a rare but uniformly rapidly progressive and fatal disease. Over the last few decades, it has been more frequently identified [7]. Presentation can be variable and may point toward an alternative diagnosis. There have been several cases reported on non-specific symptom presentation and ultimate diagnosis of CJD [8-10].

While further tests were not performed to confirm the subtype of CJD in our patient, we concluded that he suffered from the sporadic form. This is because he had no family history of CJD, nor was he known to have undertaken any neurosurgical procedures. Confirmation of CJD remains a challenge to this day [11]. The 2017 International CJD Surveillance Network diagnostic criteria for sporadic CJD (sCJD) states that the definite criteria for sCJD are progressive neurological syndrome and positive confirmatory test results (neuropathological/immunocytochemical/biochemical) [12].

MRI of the brain serves as a valuable diagnostic modality in identifying sCJD during life. A notable imaging feature, observed in this case, is the "cortical ribboning," showing areas of increased signal intensity along the cortical gyri on DWI and FLAIR sequences. This finding is commonly associated with CJD.

Electroencephalography (EEG) also plays a critical role in evaluating suspected CJD cases. Periodic sharp-wave complexes (PSWCs), i.e., triphasic waveforms typically occurring once per second in the temporal lobes, are present in approximately two-thirds of sCJD patients. This EEG pattern can be present in early stages of CJD and can make the clinician suspicious of prion disease. As the condition advances, these waveforms may become more widespread, especially in the bifrontal regions. However, it is important to note that the absence of PSWCs does not definitively rule out the disease.

CSF analysis is another part of the diagnostic process; on the contrary, it is rarely used. In the UK, CSF samples are typically analyzed at the National CJD Research & Surveillance Unit in Edinburgh. The detection of the 14-3-3 protein in CSF can point toward a diagnosis of CJD, though elevated levels can also be found in other neurological disorders such as viral encephalitis, stroke, or Hashimoto's encephalitis. Therefore, interpretation must be made in conjunction with clinical and radiological data. A more advanced technique, known as RT-QuIC, has recently emerged. This assay identifies misfolded prion proteins in the CSF and nasal brushings, offering a high diagnostic accuracy with sensitivity ranging from 80% to 90% and specificity approaching 100% in CSF.

Although considered the definitive diagnostic tool, brain biopsy is rarely performed due to several reasons. Histological confirmation requires the presence of vacuolation and detectable amyloid deposits using immunohistochemical methods, criteria not always met due to sampling limitations. Furthermore, the procedure is invasive, expensive, and generally reserved for cases where non-invasive testing is inconclusive or at the explicit request of the patient or family [13,14]. In this case, the diagnosis was supported by characteristic MRI findings and a positive RT-QuIC result [15].

There is no definitive treatment for CJD. Usually, benzodiazepines are used for treating symptoms of myoclonus, antipsychotics for psychosis or agitation, and antidepressants for mood disorders [16]. A few new treatment options have been developed. These include the use of flupirtine for cytoprotection and intraventricular pentosan polysulfate infusion. Researchers at the University College London (UCL) have also developed a monoclonal antibody, called prion protein monoclonal antibody (PRN100). However, given the small number of patients treated, researchers say the findings should be regarded as preliminary and further studies are needed to draw more comprehensive conclusions [17-19]. These are investigational drugs not used in clinical practice.

For our patient, we managed him symptomatically, with medications to deal with agitation, pain, and respiratory secretions. Unfortunately, he succumbed to the disease in four months.

Study limitations

As the patient is now deceased, any further questions that occurred to us while writing the article could not be addressed. We had to suffice with the documentation available.

## Conclusions

We have presented the case of a patient with acute confusion. His MRI findings prompted further investigations that led to a diagnosis of sporadic CJD. We wanted to raise awareness of the variable presentation of prion disease and the available diagnostic tests. This will warrant early management and hence the comfort of the patient.
